# Measuring the impact of ambulatory red blood cell transfusion on home functional status: study protocol for a pilot randomized controlled trial

**DOI:** 10.1186/s13063-017-1873-z

**Published:** 2017-03-31

**Authors:** Dennis H. Murphree, Theresa N. Kinard, Nandita Khera, Curtis B. Storlie, Che Ngufor, Sudhindra Upadhyaya, Jyotishman Pathak, Emma Fortune, Eapen K. Jacob, Rickey E. Carter, Karl A. Poterack, Daryl J. Kor

**Affiliations:** 1 Department of Health Sciences Research, Mayo Clinic, Rochester, MN USA; 2 Department of Anesthesiology, Mayo Clinic, 200 First St. SW, Rochester, MN 55905 USA; 3 Department of Anesthesiology, Mayo Clinic, 13400 E. Shea Blvd., Scottsdale, AZ 85259 USA; 4 Division of Health Informatics, Weill Cornell Medical College, 425 East 61 Street, New York, NY 10065 USA; 5 Department of Hematology, Mayo Clinic, 13400 E. Shea Blvd., Scottsdale, AZ 85259 USA; 6 Department of Pathology and Laboratory Medicine, Mayo Clinic, 13400 E. Shea Blvd., Scottsdale, AZ 85259 USA; 7 Division of Hematology, Mayo Clinic, 200 First St. SW, Rochester, MN 55905 USA

**Keywords:** Transfusion, Functional status, Outpatient, Clinical trial

## Abstract

**Background:**

Red blood cell (RBC) transfusion is frequently employed in both ambulatory and hospital environments with the aim of improving patient functional status. In the ambulatory setting, this practice is particularly common in patients with malignancy due to anemia associated with their cancer therapy. Increasingly, the efficacy of this US$10.5 billion per year practice has been called into question. While it is often standard of care for patients with chemotherapy-induced anemia to receive ambulatory RBC transfusions, it is unclear to what extent such transfusions affect home functional status. It is also unclear whether or not changes in functional status in this population can be objectively quantified using wearable activity monitors. We propose to directly measure the impact of outpatient RBC transfusions on at-home functional status by recording several physiological parameters and quantifiable physical activity metrics, e.g., daily energy expenditure and daily total step count, using the ActiGraph wGT3X-BT. This device is an accelerometer-based wearable activity monitor similar in size to a small watch and is worn at the waist. Study participants will wear the device during the course of their daily activities giving us quantifiable insight into activity levels in the home environment.

**Methods/design:**

This will be a randomized crossover pilot clinical trial with a participant study duration of 28 days. The crossover nature allows each patient to serve as their own control. Briefly, patients presenting at a tertiary medical center’s Ambulatory Infusion Center (AIC) will be randomized to either: (1) receive an RBC transfusion as scheduled (transfusion) or (2) abstain from the scheduled transfusion (no transfusion). After an appropriate washout period, participants will crossover from the transfusion arm to the no-transfusion arm or vice versa. Activity levels will be recorded continuously throughout the study using an accelerometry monitor. In addition to device data, functional status and health outcomes will be collected via a weekly telephone interview. The primary outcome measure will be daily energy expenditure. Performance metrics, such as step count changes, will also be evaluated. Additional secondary outcome measures will include daily sedentary time and Patient-reported Outcomes Measurement Information System (PROMIS) Global 10 Survey scores.

**Discussion:**

This trial will provide important information on the feasibility and utility of using accelerometry monitors to directly assess the impact of RBC transfusion on patients’ functional status. The results of the study will inform the merit and methods of a more definitive future trial evaluating the impact of ambulatory RBC transfusions in the target population.

**Trial registration:**

ClinicalTrials.gov, identifier: NCT02835937. Registered on 15 July 2016.

**Electronic supplementary material:**

The online version of this article (doi:10.1186/s13063-017-1873-z) contains supplementary material, which is available to authorized users.

## Background

The number of red blood cell (RBC) units transfused annually approaches 14 million in the United States (US) alone [1]. With an estimated cost of US$761 per RBC unit [2], this equates to US$10.5 billion in annual health care expenditures. Notably, the efficacy of RBC transfusion has been increasingly called into question [3]. This fact, when considered in concert with the underappreciated costs and risks of RBC transfusion, has resulted in a progressive move towards more conservative RBC transfusion practices [4].

Importantly, the evidence underlying these trends has primarily focused on the provision of RBC transfusion in the hospital environment. In contrast, very little data are available to guide transfusion practices in the outpatient ambulatory setting. This represents a key knowledge gap in current transfusion practice, and addressing this gap is an important goal of the proposed study.

In the ambulatory environment, RBC transfusions are frequently administered with the intention of improving patients’ functional status. Indeed, survey responses suggest that a patient’s functional status is the second leading indication for RBC transfusion trailing only the pretransfusion hemoglobin (Hgb) value [5]. However, data supporting this practice are extremely limited. A secondary outcome of the well-publicized Transfusion Trigger Trial for Functional Outcomes in Cardiovascular Patients Undergoing Surgical Hip Fracture Repair (FOCUS) specifically evaluated the impact of RBC transfusion practices on gross measures of patient’s functional status [6]. In this large clinical trial, more liberal RBC transfusion practices were not associated with improved functional status. Additional investigations have also failed to identify an association between the presence of anemia and quality of life (QoL) measures [7].

In contrast, a limited number of investigations do in fact support improved functional status with more liberal RBC transfusion practices [3, 8]. These investigations typically evaluate patient functional status in the health care environment (e.g., Timed Up and Go Test in the health care provider’s office [9]) or via survey responses to questionnaires such as the Medical Outcomes Study 36-Item Short-Form Health Survey (SF-36) [8].

Importantly, no well-designed trials have evaluated the impact of RBC transfusion on functional status in the patient’s home environment, arguably a far more relevant outcome to potential transfusion recipients. Indeed, the efficacy of RBC transfusion in the ambulatory setting remains very much a matter of debate. To this point, ongoing equipoise in this area was recently highlighted at the 2015 National Heart, Lung, and Blood Institute State of the Science Meeting in Transfusion Medicine where the identification of new strategies or technologies that may help to clarify the role of RBC transfusion in the ambulatory setting was noted to be a topic of significant interest.

Beyond unclear efficacy in the ambulatory setting, it is also important to recognize that every transfusion episode carries the potential for meaningful risk. As examples, transfusion-related acute lung injury (TRALI) and transfusion-associated circulatory overload (TACO) are respiratory complications resulting from transfusion and are the leading causes of transfusion-related death in the US [10]. Although great strides have recently been made in predicting patient-specific risk prior to transfusion [11–13], the risk remains serious. Furthermore, despite their significance, patients and their clinical teams frequently fail to recognize these transfusion-related respiratory complications [14–16]. Compounding the risk landscape is the consideration that at present there are essentially no data evaluating the risks of transfusion in the ambulatory setting.

The introduction of innovative mobile health (mHealth) technologies creates unique opportunities to study patients’ functional status in a more robust manner and in the setting that matters most: their home. Indeed, as the accuracy of these novel devices improves, their potential application to studying outcomes in the home environment moves closer and closer to a reality.

### Aims and objectives

#### Primary

In light of the ongoing equipoise regarding the efficacy of RBC transfusion in the ambulatory setting, the known risks associated with blood product administration, and the availability of innovative technologies that are able help to address these concerns, the primary aims of our proposed protocol are as follows:

- **Specific aim 1**: to evaluate the impact of RBC transfusion on home functional status as assessed by physical activity metrics recorded by a wearable ActiGraph wGT3X-BT mHealth device. We hypothesize that the percent change in mean daily physical activity in the 7 days following randomization will be greater during the study periods where participants received an RBC transfusion than during the periods where an RBC transfusion was not received.
- **Specific aim 2**: to evaluate the correlation between physical activity as assessed by the accelerometer and subjective functional status as assessed with the Patient-reported Outcomes Measurement Information System (PROMIS) Global 10 Survey. We hypothesize that there will be a direct correlation between physical activity as assessed by the device and the subjective functional status as assessed by the PROMIS Global 10 Survey.

### Secondary

As a pilot trial we further delineate a set of secondary objectives. These are less formal than our primary objectives and are designed to gauge study feasibility, with a particular focus on scalability of methods to a potentially larger future trial. To that end we aim to evaluate the suitability of the ActiGraph wGT3X-BT for use in our target population. While the device has been validated in terms of its technical measurement [17–22], it remains an open question as to whether or not our patient population, most of whom are receiving chemotherapy or are transplant recipients, will display a level of adherence that is sufficient for answering our larger clinical question. We will also monitor enrollment volume, trial completion volume, device failure rates, and other such measures which might be of practical importance when considering scaling to a larger future trial.

In order to consider whether or not to move forward from a pilot trial to a larger future randomized clinical trial, we will carefully evaluate the results for a signal of efficacy. If there is strong evidence that RBC transfusion is efficacious in improving patient activity levels a larger trial is less likely to be considered. Alternatively, if there is evidence that “no transfusion” is better, or if the results are equivocal, we are more likely to pursue a larger trial. In addition to intervention efficacy, we will also assess both the feasibility of scaling the trial to a more definitive phase II/III design and the impact of any adverse events. We will carefully consider participant adherence rate and the potential for increasing enrollment as prerequisites for proceeding to a larger trial. Adverse events, if any, will be evaluated as outlined by the Safety and Monitoring Plan (Additional file 1). The final document reporting the results of this trial will follow the main Consolidated Standards of Reporting Trials (CONSORT) 2010 Statement: extension to randomized pilot and feasibility trials [23]. Both this trial and any future trial will follow the Standard Protocol Items: Recommendations for Interventional Trials (SPIRIT) Checklist (Additional file 2).

## Methods/design

### Study design

**Fig. 1:**
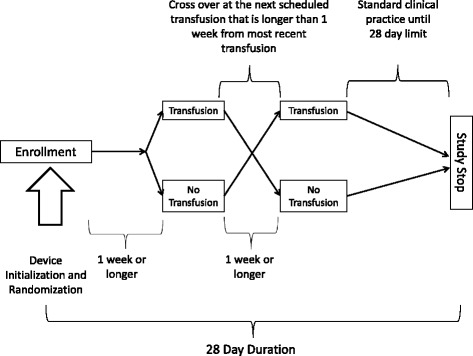
Study design

This will be a randomized, two-group, crossover, pilot clinical trial (Fig. 1). Following this design each subject serves as their own control. Randomization is to help eliminate bias due to any time-dependent change in subject health or chronological effects that may result from a nontransfusion period.

### Participants and screening

Study participants will be identified in the Ambulatory Infusion Center (AIC) of a tertiary care medical center. The study population will consist of adult (aged 18 years or older) male and female hematology and oncology patients presenting to the AIC with anemia (Hgb under practice standard threshold for transfusion, e.g., 7.0–9.0 g/dL) and who are planned to undergo routine RBC transfusion. Subjects with at least one prior presentation to the AIC within 3 months of the screening visit will be eligible for study participation. This inclusion criterion has been selected due to the fact that our preliminary research indicates that those who present to the AIC on more than one occasion for RBC transfusion are highly likely to present for a subsequent RBC transfusion in a 28-day time frame. A full list of the inclusion and exclusion criteria are provided in Table 1.

**Table 1:** Inclusion and exclusion criteria

Inclusion criteria	Exclusion criteria
Age ≥18 years	Refusal to provide informed consent
At least one prior encounter in the AIC	Refusal by the health care team
Planned RBC transfusion	Acute ischemia (e.g., myocardial infarction (MI), cerebrovascular accident (CVA))
	Hgb <7.0 g/dL
	Active bleeding
	Symptomatic anemia (hypotension, tachycardia, angina, syncope/presyncope believed related to anemia)
	Nonambulatory functional status
	Established or uncertain pregnancy status

*AIC* Ambulatory Infusion Center, *RBC* red blood cell

For the exclusion criteria of pregnancy, potential study participants in the reproductive age group will be asked if they might be pregnant. If the response is “yes” or “unknown,” the potential study participant will be excluded. Screening/consent logs will be maintained centrally to allow generation of a CONSORT diagram, but will also made available to the transfusion medicine service at Mayo Clinic in Phoenix, AZ, USA to facilitate the procedures outlined below. Consenting participants will be provided with an accelerometer to wear continuously for the duration of their involvement in the trial (28 days). This device, described further below, will automatically capture relevant physiological data with no need for intervention by the patient. Following the trial, participants return the device by mail and data are retrieved by the investigative team.

The accelerometer chosen for this study is the ActiGraph wGT3X-BT (ActiGraph Corp, Pensacola, FL, USA). These devices are similar in size and weight to a simple wristwatch, and are typically worn at the wrist, ankle or waist. By capturing and interpreting acceleration data the device objectively measures a patient’s physical activity through metrics such as step counts, energy expenditure, daily metabolic equivalent (MET) rates, activity intensity, activity bouts, and sedentary bouts. For the current study, the devices will be worn at the waist in consideration of the more extensive peer review given to waist-based algorithms used to interpret raw accelerometer data. Additional measurable quantities include wear time, heart rate, RR intervals, and body position.

All study participants will be contacted weekly via telephone for the duration of the study (expected to be 28 days). The purpose of these communications is to better understand wearability, enhance protocol compliance, and assess satisfaction with the device. The Data Capture Form used by the study coordinator (SC) is available as supplemental material to this article (Additional file 3). All comments by participants will be logged by the SC, giving the team insight into participant interaction with the device and protocol. Inquiries into life events that may impact home functional status (e.g., injuries, illnesses, other events) will also be made. Finally, symptoms related to the intervention of interest (transfusion versus no transfusion) will also be assessed during each of these correspondences. Specific inquires will include evidence of profound anemia, renal failure, myocardial infarction, nonhemorrhagic stroke, mesenteric ischemia, syncope, falls, shortness of breath, and chest pain or pressure as well as any medical encounters including nonstudy transfusion events. All responses will be augmented with data recorded in participants’ electronic health records.

### Randomization

After informed consent has been obtained, study participants will be stratified into those that are actively receiving chemotherapy and those that are not. Within each stratum, participants will be randomized using simple randomization (Pr(transfusion group first) = 0.5). Patients successfully enrolled will be randomized to one of two transfusion/no transfusion sequences (Fig. 1). The SC will obtain the randomized sequence from the Research Electronic and Data Capture (REDCap) randomization module. After randomization, the SC will inform the AIC of the appropriate timing of the no-transfusion study period so that an updated order set can be prepared. It is recognized that using simple randomization may result in imbalances in the transfusion sequences, but simple randomization was selected for this study for ease of implementation. The crossover design also helps mitigate confounding provided that there is no period effect observed in the data.

### Intervention

Those randomized to transfusion will receive the standard-of-care-ordered RBC transfusion. This means that they will receive the transfusion as specifically ordered by their primary physician, typically one to two units of allogeneic RBCs. Those randomized to the no-transfusion arm will be discharged from the AIC without receiving an RBC transfusion. The patients who are randomized into the transfusion group will be crossed over into the no-transfusion group and the patients who are randomized into the no-transfusion group will be crossed over into the transfusion group at a subsequent visit. The time of this crossover for a given patient will be at their next scheduled transfusion that is no less than 1 week from the time of their most recent RBC transfusion in this trial.

### Blinding

Due to the nature of the intervention (transfusion versus no transfusion), blinding to the intervention will not be feasible for either the patient or the clinical team in this pilot clinical trial. The accelerometer does not provide any visual indication of activity levels (e.g., no step count or estimated energy expenditures), so participants will be blinded to the data accumulated by the device. With the exception of the Data Monitoring Committee, investigators will not have access to the accelerometer data until the completion of the trial. Patients may access their data after completion of the trial by request to the study team. Because blinding to the intervention is impossible, and because the purpose of the trial is carefully explained to participants beforehand, we note the potential for both performance and detection biases. In particular, many participants may have an expectation of lower functional status after a no-transfusion encounter. One downstream consequence of this is a potential bias towards lack of effect, meaning that transfusions may be biased to appear more effective than the no-transfusion intervention. Concerns related to selection bias are expected to be largely mitigated by randomization and by the crossover nature of the design. Nonetheless, risks related to residual biases resulting from unique characteristics associated with patients who consent to participate in this trial clearly remain.

### Measures

The primary outcome measure of this study is the estimated daily energy expenditure using, e.g., the Williams Work-Energy Approximation calculated from acceleration-based activity counts and body weight. We choose this metric because it has been shown to be correlated with energy expenditure estimations from indirect calorimetry, is a clear indicator of activity level and has been well studied in the medical literature [17, 19–22, 24]. We emphasize that our study design seeks to measure changes in activity levels, thus the absolute accuracy of the energy expended by a participant is not critical to our broader clinical question. Secondary outcome measures include daily step count, daily sedentary time, MET rate, and PROMIS Global 10 Survey results. This survey is a ten-question assessment designed to measure overall physical, mental, and social health as well as pain, fatigue, and perceived QoL. Further exploratory outcome measures will consider activity bout counts, estimated energy expenditure on a more finely resolved time scale (e.g., minute to minute), nonintervention RBC transfusions, and need for hospital admission or other health care encounters.

### Data collection and management

#### Sources and repositories

There are five sources of data in this trial – base data collected by the devices, derived data interpreted by the device analysis software, weekly survey data collected via telephone, study participation data such as Consent Forms, and finally, encounter-associated data such as SC encounter notes or laboratory results measured in the AIC.

During the enrollment and data collection phases of the study, the primary data repository will be a set of hard copy study participant files that will be maintained by the lead SC at the AIC. A centralized digital data store (CDS) will also be maintained at an access-restricted and access-logged study site within Mayo Clinic’s secure network. As the study progresses and patient data are recovered from devices, this raw device data will be added to the CDS. In order to facilitate ease of interpretation and analysis after collection, the CDS will also be tied to a REDCap store. Only those with approved access to the REDCap data management system will have access to the study data. Telephone survey and AIC encounter data will also be transferred from the hard copy files into REDCap by the SC.

During the analysis phase, all derived data and global study result data will also be stored in the CDS. Upon study data collection completion, all relevant physical files not yet present in the CDS will be scanned and integrated into the digital store. Physical files will be held and then disposed of according to the Mayo Clinic record retention policy. The primary repository of data for analysis and reporting will thus be the CDS.

### Practice logistics and workflow

A critical component for the successful completion of this study is close integration with the current hematology and oncology practices at the AIC. From a practical perspective, engineering this integration has been the most challenging aspect of moving the study forward. Because of this, we detail our practice logistics and workflow model.

It is important to remember that in this protocol *not* transfusing the patient is the intervention – otherwise standard practice is followed. Thus, from a practice logistics and patient safety point of view it is during the no-transfusion arm that special care must be taken.

The current practice at the AIC is for patients receiving transfusion therapies (e.g., chemotherapy) to be associated with a set of standing orders by their practicing physician. These standing orders are typically directions to transfuse the patient when their Hgb is measured below a specific level. For many practicing physicians this Hgb level will be between 7.0 and 9.0 g/dL. These standing orders are specified for a defined time period up to 3 months so their modification is an important aspect of implementing the proposed protocol.

When a SC identifies a patient who appears to be a likely candidate, they will contact the potential study participant’s primary physician to receive conditional approval for inclusion in the study. Patients receiving conditional provider approval will have Hgb levels monitored on the day of their AIC visit by the SC. If the Hgb is within the range prescribed for transfusion, the patient is approached for screening and informed consent. If enrolled, the SC will randomize the participant. If the participant’s next encounter is to transfuse, no change to the standing orders is required.

If the patient’s next encounter is no-transfusion, new standing orders are requested. These orders are designed to avoid RBC transfusion unless specific “over-riding” RBC transfusion criteria are present. These “over-riding” RBC transfusion criteria include: acute ischemia (e.g., MI, CVA), Hgb <7.0 g/dL, active bleeding, and symptomatic anemia (hypotension, tachycardia, angina or syncope/presyncope believed related to anemia). If none of the above mentioned “over-riding” RBC transfusion criteria are present, the patient will not be transfused.

After the participant crosses from one study arm to the other, the SC requests new standing orders as necessary.

### Data monitoring

For this trial, data monitoring consists of measures required for patient privacy and to ensure the success of the trial. Privacy compliance will follow the standards set by both the Health Insurance Portability and Accountability Act (HIPAA) and by Mayo Clinic internal standards. To help ensure the accuracy of the study data, the Data Monitoring Committee of Drs. Murphree and Kor will randomly sample and review the validity of data collected, especially device data. Throughout the trial ongoing assessment will monitor for device data loss, partial study completion, and other potentially unforeseen data concerns. In the event of data loss due to partial study completion, the patient will be removed from the cohort. In the event of data loss due to device issues or other unforeseen events the impact of the loss will be assessed by the investigators. If the loss compromises the integrity of the study the patient will be removed from the cohort. A valid device wear hour is defined as ≤30 min of consecutive “zero” values (no activity) and a valid day as ≥10 wear hours per day [25]. If the loss is consistent with reasonably acceptable missing data; for example, the subject forgetting to put the device on for an hour after bathing, then the missing data will be dealt with via appropriate statistical methods. These could include amongst others a Bayesian-based imputation or an all-available average, with the appropriate method chosen based on the nature of the missingness. Care will be taken to insure consistency across subjects and missing-data episodes.

### Statistics

#### General descriptive statistics and preliminary data screening

Baseline demographics, clinical characteristics, and procedure-related information will be presented as median (25–75% interquartile range (IQR)) for continuous data elements and frequencies (%) for categorical data.

Accelerometer data will be preprocessed by the ActiLife [26] software to convert the raw accelerometer signal into expressions of movement and energy expenditure. These calculations require some basic patient information (e.g., weight), so the device configuration will be verified prior to finalization of the calculations. For the primary analysis, we will aggregate the summaries on a daily basis. For exploratory analysis we will use the summaries on a finer time resolution (e.g., minute to minute) to determine whether this provides a more sensitive method of detecting subtle changes in activity levels.

### Primary analysis

The primary outcome analyses will be performed using an “as treated” rather than an “intention-to-treat” analysis set while accounting for the crossover design. This approach is most appropriate for this pilot clinical trial, which primarily aims to understand home functional status as it relates to ambulatory RBC transfusion. For statistical comparisons, we will fit a random effects model that utilizes daily total energy expenditure as the dependent variable and days from transfusion or control visit (“placebo” visit where no transfusion was performed). For simplicity, we will include the mean daily measurements for the 3 days prior to the transfusion/control visit as covariates in the model, and consider the 7 days after transfusion/control visit in the model. This censoring of data beyond 7 days after the transfusion/control visit is to avoid carryover effects with the model. We will test the transfusion indicator by study day interaction, with adjustment for baseline activity, for the primary hypothesis. This parameter will quantify how the profile of activity differs with and without transfusion while accounting for the variation in activity between participants by means of the random effect and baseline adjustment. Contrasts will be constructed to quantify the difference in the change in activity between these visits.

Sensitivity analyses will attempt to model the change in activity levels in the days after transfusion. These exploratory models will consider a potential initial boost in activity with linear or exponential trends to account for the change in activity over the 7 days after transfusion. These models will also include subject random effect and account for time-series correlation. These analyses will be expanded to include all data measured over the 28-day period.

### Secondary endpoint analysis

Secondary endpoints measured by the accelerometer will be modeled similarly to the primary outcome measure. The association of the PROMIS Global 10 Survey score and activity will be quantified by the rank order correlation of the area under the curve for the incremental gain in activity over the 7 days (mean of the 7 days after transfusion/control visit minus the mean of the 3 days prior to the transfusion/control visit) and the PROMIS Global 10 Survey total score. We will conduct this on the entire data (transfusion and control visits) as well as with a partial correlation controlling for whether a transfusion was actually conducted.

### Safety analysis

Adverse events will be captured and summarized. These events will be attributable to whether or not they were transfusion-related or related to withholding the transfusion. The Data and Safety Monitoring Plan submitted to the Mayo Clinic Institutional Review Board (IRB) includes definitions of adverse events and reporting requirements. This monitoring plan is also available as supplemental material to this article. No formal stopping rules for safety are proposed, so standard descriptive summaries will be tabulated.

### Exploratory analyses

Predictor variables other than the intervention, including pertinent baseline demographics and clinical characteristics such as age, sex, race, comorbidities, medications (including chemotherapy regimens), and pretransfusion functional status, will be recorded. Additional predictor variables will include vital signs and laboratory values that are obtained during the course of routine care. Responses from the weekly telephone communications will also be recorded for potential analysis.

### Sample size considerations and recruitment

This is a pilot study to determine whether accelerometers are sensitive enough to detect change in functional status in patients undergoing ambulatory transfusion services. The primary hypothesis is to detect change in total energy expenditure after the transfusion in both between and within individual comparisons. A minimum clinically relevant difference is not yet known for this outcome. To estimate the sample size, we approach the calculation from the perspective of having sufficient data to estimate the standard deviation so that we could plan a follow-up study. Julious [27] recommends studying 12 participants per group for such aims. We apply this recommendation to this study by assuming that we want at least 12 participants to complete the first randomized phase of the crossover design (i.e., the parallel group portion of the study). Based on this, we will want to have at least 24 subjects who will provide evaluable data from the activity monitor. We therefore adjust the sample size upwards by 25% to account for attrition and/or changes in the transfusion schedule. Thus, our total planned sample size is 30 participants. Preliminary research with clinicians familiar with the infusion center patient population suggests that an enrollment of this size should be readily achievable. Specific strategies for achieving adequate enrollment include presentations to the hematology and oncology practices in order to advertise the study to providers, as well as access to the Mayo Clinic patient scheduling and electronic health record system. This will ensure the ability to have sufficient on-site recruiting staff on days where patients known to be eligible are scheduled for transfusion.

## Discussion

Blood product transfusion is a frequently employed medical practice, with an estimated 5 million patients [28] annually receiving blood in the US alone. However, despite its relative ubiquity, practitioners are increasingly questioning the utility of the practice. Concern stems from three sources – the recognition of risks associated with RBC transfusion, the uncertain efficacy in improving patient outcomes, and the expense of this limited resource.

In terms of efficacy in improving patient outcomes, the landscape is complicated. While it is well documented that severe anemia increases a patient’s risk of mortality [29–33], it is often unclear whether correcting the anemia results in an improved outcome [34, 35]. While there is growing consensus that blood transfusions save lives for some of the sickest patients (e.g., those with hemorrhagic shock), for many patients, a transfusion may cause more harm than good. Recent work examining the effects of more restrictive transfusion practices has found significant improvement in patient outcomes across all hospital environments [36], and a large number of studies document allogeneic transfusion as being associated with a variety of adverse surgical outcomes as well as adverse clinical outcomes, particularly for cancer patients [37–45].

There is a striking lack of data regarding transfusion in ambulatory patient populations. This current work is particularly relevant because no published studies have examined an optimal transfusion threshold for ambulatory patients. The current state of knowledge regarding transfusion efficacy has been acquired from studies of hospitalized patients.

Some work has been done examining the effects of transfusion on chronically anemic populations, specifically those with myelodysplastic syndromes (MDS) [46]. One small study [47] found that treating patients to a target Hgb of 12.0 g/dL by either darbepoetin alfa injection or via RBC transfusion resulted in significant improvement in QoL from both treatments. If replicated at scale, this might perhaps suggest modifying transfusion practice for this population. More generally, a recent medical literature review [48] concludes that there is currently a lack of evidence to support a particular transfusion strategy for patients with MDS and calls for robust randomized trials. There are currently two ongoing clinical trials seeking an optimal transfusion threshold for improving QoL in patients with MDS: ClinicalTrials.gov **#**NCT02099669 and ISRCTN #26088319. Both are to complete in 2016 and are examining QoL effects of liberal versus restrictive transfusion strategies.

Limited work has also been done considering late-stage cancer patients in a palliative care setting, with two studies [49, 50] from the late 1990s indicating relief of several symptoms. In a more recent review [51], the authors found evidence of relief for fatigue and breathlessness, while also noting a significant proportion of participants dying within 2 weeks of transfusion. These investigations further highlighted the need for higher-quality future studies.

The dangers of RBC transfusion can fall into two categories: immunological and nonimmunological transfusion reactions. Life-threatening immunological transfusion reactions include anaphylaxis, acute hemolysis and TRALI. Nonimmunological transfusion reactions that contribute to morbidity and mortality include TACO and transfusion-transmitted infection. Long-term risks of transfusion to a chronically anemic patient include iron overload which may result in multiorgan damage. While some risks have been dramatically reduced, many risks continue to persist. For example, transfusion transmission of the well-characterized viruses, such has hepatitis C virus and human immunodeficiency virus, has been significantly reduced by implementing donor screening, while other potential transfusion-transmitted infections remain a moving target and require constant vigilance. The recent emergence of Zika virus in the US and corresponding reactions by the Center for Disease Control and AABB illustrates this particular challenge.

In addition to the above concerns about efficacy and safety, it is important to remember that blood products are a precious resource. Considering data from 2011 [1], the available supply of whole blood/RBC units exceeded the number of transfused units by only 5.2%, with a trend in donation rates dropping 3–4% per year. An aging US population can only be expected to exacerbate the situation. Furthermore, the direct and indirect costs associated with blood product transfusion are rising [52]. Goodnough et al. [36] found that over a 5-year period, implementing a best-practice alert triggered for higher Hgb transfusions saved a large hospital US$1.3 million annually in purchasing costs alone.

With the above in mind, it is clear that deeper understanding of the true utility of RBC transfusion is of vital importance to the field. The randomized crossover clinical trial proposed here aims to provide preliminary information on the feasibility of measuring the efficacy of RBC transfusion in improving home functional status with a wearable activity monitor. The study, while it can be described as a pilot, is robust in that it utilizes a randomized crossover design, incorporates an objective measurement of activity based on accelerometer data, and includes patient-reported health status. This study will provide novel data that will aid in the elucidation of transfusion’s influence on ambulatory activity following outpatient transfusion.

In addition to the strengths of the trial noted above, we also acknowledge potential weaknesses of the protocol. Such limitations include concerns relating to the detectability of activity level in what may often be a sedentary population with variable daily activity levels, risk that the effect from transfusion may exceed the 1-week washout period, and difficulty in recruiting patients who will be willing to consistently wear a monitor for 28 days. Though we acknowledge these potential challenges, the insights gleaned will greatly facilitate the potential design and conduct of a future, more definitive, clinical trial. We look forward to seeing the results of this trial and to gaining important insight into this clinically relevant question.

### Current study status

Patient recruitment began in August 2016 and is due to be completed in December 2017.

## Additional files

Additional file 1: Safety and Monitoring Plan Supplement. (DOCX 132 kb)

Additional file 2: SPIRIT 2013: SPIRIT (Standard Protocol Items: Recommendations for Interventional Trials) Checklist for clinical trial protocols. (DOCX 65 kb)

Additional file 3: Telephone assessment Data Capture Form. (PDF 12 kb)

## Abbreviations

AIC: Ambulatory Infusion Center
CDS: Centralized digital data store
CVA: Cerebrovascular accident
Hgb: Hemoglobin
HIPAA: Health Insurance Portability and Accountability Act
IRB: Institutional Review Board
MDS: Myelodysplastic syndromes
mHealth: Mobile health
MI: Myocardial infarction
PROMIS: Patient-reported Outcomes Measurement Information System
QoL: Quality of life
RBC: Red blood cell
REDCap: Research Electronic and Data Capture
SC: Study coordinator
TACO: Transfusion-associated circulatory overload
TRALI: Transfusion-related acute lung injury
US: United States

## References

[1] The 2011 National Blood Collection and Utilization Survey Report. https://www.hhs.gov/sites/default/files/ash/bloodsafety/2011-nbcus.pdf. Accessed 7 June 2016.

[2] Shander A, Hofmann A, Ozawa S, Theusinger OM, Gombotz H, Spahn DR, SABM, MSBM. Activity-based costs of blood transfusions in surgical patients at four hospitals. Transfusion. 2010;50:753–65. 10.1111/j.1537-2995.2009.02518.x 20003061

[3] Nielsen K, Johansson PI, Dahl B, Wagner M, Frausing B, Borglum J, Jensen K, Sturup J, Hvolris J, Rasmussen LS. Perioperative transfusion threshold and ambulation after hip revision surgery—a randomized trial. BMC Anesthesiol. 2014;14. DOI: 10.1186/1471-2253-14-89. 10.1186/1471-2253-14-89 PMC4203913 25337035

[4] Carson JL, Guyatt G, Heddle NM, Grossman BJ, Cohn CS, Fung MK, Gernsheimer T, Holcomb JB, Kaplan LJ, Katz LM (2016). Clinical practice guidelines from the AABB: red blood cell transfusion thresholds and storage. JAMA.

[5] Whitman CB, Shreay S, Gitlin M, van Oijen MG, Spiegel BM (2013). Clinical factors and the decision to transfuse chronic dialysis patients. Clin J Am Soc Nephrol.

[6] Carson JL, Terrin ML, Noveck H, Sanders DW, Chaitman BR, Rhoads GG, Nemo G, Dragert K, Beaupre L, Hildebrand K (2011). Liberal or restrictive transfusion in high-risk patients after hip surgery. N Engl J Med.

[7] So-Osman C, Nelissen R, Brand R, Brand A, Stiggelbout AM (2011). Postoperative anemia after joint replacement surgery is not related to quality of life during the first two weeks postoperatively. Transfusion.

[8] Conlon NP, Bale EP, Herbison GP, McCarroll M (2008). Postoperative anemia and quality of life after primary hip arthroplasty in patients over 65 years old. Anesth Analg.

[9] Wallis JP, Wells AW, Whitehead S, Brewster N (2005). Recovery from post-operative anaemia. Transfus Med.

[10] Fatalities Reported to FDA Following Blood Collection and Transfusion: Annual Summary for Fiscal Year 2014. https://www.fda.gov/downloads/BiologicsBloodVaccines/SafetyAvailability/ReportaProblem/TransfusionDonationFatalities/UCM459461.pdf. Accessed 1 June 2016.

[11] Murphree D, Ngufor C, Upadhyaya S, Madde N, Clifford L, Kor DJ, Pathak J (2015). Ensemble learning approaches to predicting complications of blood transfusion. Conf Proc IEEE Eng Med Biol Soc.

[12] Murphree DH, Clifford L, Lin Y, Madde N, Ngufor C, Upadhyaya S, Pathak J, Kor DJ. Predicting adverse reactions to blood transfusion. In: Healthcare Informatics (ICHI), 2015 International Conference: 21–23 Oct 2015. 2015: 82–89.

[13] Murphree DH, Clifford L, Lin Y, Madde N, Ngufor C, Upadhyaya S, Pathak J, Kor DJ. A clinical decision support system for preventing adverse reactions to blood transfusion. In: Healthcare Informatics (ICHI), 2015 International Conference: 21–3 Oct 2015. 2015: 100–104.

[14] Clifford L, Singh A, Wilson GA, Toy P, Gajic O, Malinchoc M, Herasevich V, Pathak J, Kor DJ (2013). Electronic health record surveillance algorithms facilitate the detection of transfusion-related pulmonary complications. Transfusion.

[15] Kopko PM, Marshall CS, MacKenzie MR, Holland PV, Popovsky MA (2002). Transfusion-related acute lung injury: report of a clinical look-back investigation. JAMA.

[16] Narick C, Triulzi DJ, Yazer MH (2012). Transfusion-associated circulatory overload after plasma transfusion. Transfusion.

[17] McMinn D, Acharya R, Rowe DA, Gray SR, Allan JL (2013). Measuring activity energy expenditure: accuracy of the GT3X+ and actiheart monitors. Int J Exerc Sci.

[18] Fortune E, Lugade VA, Amin S, Kaufman KR (2015). Step detection using multi-versus single tri-axial accelerometer-based systems. Physiol Meas.

[19] Jarrett H, Fitzgerald L, Routen AC (2015). Interinstrument reliability of the ActiGraph GT3X+ ambulatory activity monitor during free-living conditions in adults. J Phys Act Health.

[20] Johnson MJ, Turek J, Dornfeld C, Drews J, Hansen N (2016). Energy expenditure and step count accuracy of the Actigraph wGT3X-BT during walking and running: 1265 Board #4 June 2, 8:00 AM–10:00 AM. Med Sci Sports Exerc.

[21] Santos-Lozano A, Santin-Medeiros F, Cardon G, Torres-Luque G, Bailon R, Bergmeir C, Ruiz JR, Lucia A, Garatachea N (2013). Actigraph GT3X: validation and determination of physical activity intensity cut points. Int J Sports Med.

[22] Westerterp KR (2014). Reliable assessment of physical activity in disease: an update on activity monitors. Curr Opin Clin Nutr Metab Care.

[23] Eldridge SM, Chan CL, Campbell MJ, Bond CM, Hopewell S, Thabane L, Lancaster GA, PAFS Consensus Group (2016). CONSORT 2010 Statement: extension to randomised pilot and feasibility trials. BMJ.

[24] Fortune E, Tierney M, Scanaill CN, Bourke A, Kennedy N, Nelson J. Activity level classification algorithm using SHIMMER wearable sensors for individuals with rheumatoid arthritis. Conference proceedings: Annual International Conference of the IEEE Engineering in Medicine and Biology Society IEEE Engineering in Medicine and Biology Society Annual Conference 2011, 2011:3059–3062. 10.1109/IEMBS.2011.6090836 22254985

[25] Troiano RP, Berrigan D, Dodd KW, Masse LC, Tilert T, McDowell M (2008). Physical activity in the United States measured by accelerometer. Med Sci Sports Exerc.

[26] ActiGraph: ActiLife. vol. http://www.actigraphcorp.com/support/software/actilife/. Accessed 7 June 2016.

[27] Julious SA (2005). Sample size of 12 per group rule of thumb for a pilot study. Pharm Stat.

[28] Sharma S, Sharma P, Tyler LN (2011). Transfusion of blood and blood products: indications and complications. Am Fam Physician.

[29] Carson JL (1996). Effect of anaemia and cardiovascular disease on surgical mortality and morbidity. Lancet.

[30] Carson JL, Noveck H, Berlin JA, Gould SA (2002). Mortality and morbidity in patients with very low postoperative Hb levels who decline blood transfusion. Transfusion.

[31] Carson JL, Sieber F, Cook DR, Hoover DR, Noveck H, Chaitman BR, Fleisher L, Beaupre L, Macaulay W, Rhoads GG (2015). Liberal versus restrictive blood transfusion strategy: 3-year survival and cause of death results from the FOCUS randomised controlled trial. Lancet.

[32] Shander A, Javidroozi M, Naqvi S, Aregbeyen O, Caylan M, Demir S, Juhl A (2014). An update on mortality and morbidity in patients with very low postoperative hemoglobin levels who decline blood transfusion. Transfusion.

[33] Wu WC, Schifftner TL, Henderson WG, Eaton CB, Poses RM, Uttley G, Sharma SC, Vezeridis M, Khuri SF, Friedmann PD (2007). Preoperative hematocrit levels and postoperative outcomes in older patients undergoing noncardiac surgery. JAMA.

[34] Shander A, Javidroozi M, Ozawa S, Hare GMT (2011). What is really dangerous: anaemia or transfusion?. Br J Anaesth.

[35] Hare GMT, Freedman J, David MC (2013). Review article: risks of anemia and related management strategies: can perioperative blood management improve patient safety?. Can J Anesth.

[36] Goodnough LT, Maggio P, Hadhazy E, Shieh L, Hernandez-Boussard T, Khari P, Shah N (2014). Restrictive blood transfusion practices are associated with improved patient outcomes. Transfusion.

[37] Acheson AG, Brookes MJ, Spahn DR (2012). Effects of allogeneic red blood cell transfusions on clinical outcomes in patients undergoing colorectal cancer surgery. A systematic review and meta-analysis. Ann Surg.

[38] Carson JL, Carless PA, Hebert PC. Transfusion thresholds and other strategies for guiding allogeneic red blood cell transfusion. The Cochrane database of systematic reviews. 2012;4:CD002042. doi: 10.1002/14651858.CD002042.pub3. 10.1002/14651858.CD002042.pub3 PMC4171966 22513904

[39] Glance LG, Dick AW, Mukamel DB, Fleming FJ, Zollo RA, Wissler R, Salloum R, Meredith UW, Osler TM (2011). Association between intraoperative blood transfusion and mortality and morbidity in patients undergoing noncardiac surgery. Anesthesiology.

[40] Hopewell S, Omar O, Hyde C, Yu LM, Doree C, Murphy MF. A systematic review of the effect of red blood cell transfusion on mortality: evidence from large-scale observational studies published between 2006 and 2010. BMJ Open. 2013;3. PMID: 23645909, PMCID: PMC3646177. DOI: 10.1136/bmjopen-2012-002154. 10.1136/bmjopen-2012-002154 PMC3646177 23645909

[41] Koch CG, Li L, Sessler DI, Figueroa P, Hoeltge GA, Mihaljevic T, Blackstone EH (2008). Duration of red-cell storage and complications after cardiac surgery. N Engl J Med.

[42] Liu L, Wang ZW, Jiang SQ, Shao BF, Liu JB, Zhang SQ, Zhou YL, Zhou Y, Zhang YX: Perioperative allogenenic blood transfusion is associated with worse clinical outcomes for hepatocellular carcinoma: a meta-analysis. PLoS One. 2013;8. doi: 10.1371/journal.pone.0064261. 10.1371/journal.pone.0064261 PMC3669337 23741309

[43] Perkins HA, Busch MP (2010). Transfusion-associated infections: 50 years of relentless challenges and remarkable progress. Transfusion.

[44] Schiergens TS, Rentsch M, Kasparek MS, Frenes K, Jauch KW, Thasler WE (2015). Impact of perioperative allogeneic red blood cell transfusion on recurrence and overall survival after resection of colorectal liver metastases. Dis Colon Rectum.

[45] Villanueva C, Colomo A, Bosch A, Concepcion M, Hernandez-Gea V, Aracil C, Graupera I, Poca M, Alvarez-Urturi C, Gordillo J (2013). Transfusion strategies for acute upper gastrointestinal bleeding. N Engl J Med.

[46] Koutsavlis I (2016). Transfusion thresholds, quality of life, and current approaches in myelodysplastic syndromes. Anemia.

[47] Nilsson-Ehle H, Birgegard G, Samuelsson J, Antunovic P, Astermark J, Garelius H, Engstrom LM, Kjeldsen L, Nilsson L, Olsson A (2011). Quality of life, physical function and MRI T2* in elderly low-risk MDS patients treated to a haemoglobin level of ≥120 g/L with darbepoetin alfa ± filgrastim or erythrocyte transfusions. Eur J Haematol.

[48] Gu Y, Estcourt LJ, Doree C, Trivella M, Hopewell S, Vyas P. Comparison of a restrictive versus liberal red cell transfusion policy for patients with myelodysplasia, aplastic anaemia, and other congenital bone marrow failure disorders. The Cochrane database of systematic reviews. 2015;3:CD011577. doi: 10.1002/14651858.CD011577. 10.1002/14651858.CD011577 PMC4430822 25983657

[49] Sciortino AD, Carlton DC, Axelrod A, Eng M, Zhukovsky DS, Vinciguerra V (1993). The efficacy of administering blood transfusions at home to terminally ill cancer patients. J Palliat Care.

[50] Gleeson C, Spencer D (1995). Blood-transfusion and its benefits in palliative care. Palliat Med.

[51] Preston NJ, Hurlow A, Brine J, Bennett MI. Blood transfusions for anaemia in patients with advanced cancer. Cochrane Database Syst Rev. 2012 Feb 15;(2):CD009007. doi: 10.1002/14651858.CD009007.pub2. 10.1002/14651858.CD009007.pub2 PMC7388847 22336857

[52] Shander A, Hofmann A, Gombotz H, Theusinger OM, Spahn DR (2007). Estimating the cost of blood: past, present, and future directions. Best Pract Res Clin Anaesthesiol.

