# Supplementary material for: Measuring the impact of ambulatory red blood cell transfusion on home functional status: study protocol for a pilot randomized controlled trial
Source: Trials. 2017 Mar 31;18:153. doi: 10.1186/s13063-017-1873-z (PMC5374599; doi:10.1186/s13063-017-1873-z)
Supplement: Supplementary file 1 — Safety and Monitoring Plan Supplement. (DOCX 132 kb) [file 13063_2017_1873_MOESM1_ESM.docx]

C.5. Data Safety and Monitoring Plan:

Data quality and patient safety will be monitored by the PI’s of this protocol (Kor, Poterack, and Murphree). Due to the small pilot nature of this investigation and clear equipoise regarding the efficacy and risk of RBC transfusions in the setting of this protocol, an external Data Safety and Monitoring Board is not planned. The PIs will continuously monitor the data quality according to standard data quality measures. In addition, recruitment rate, deviation from inclusion/exclusion criteria and protocol, and confidentiality of data will be monitored. Adverse events will be monitored by the site PIs and research specialists in real time from the start of participant enrollment until completion of the study protocol (28 days for each study participant). Adverse events will be defined as ‘unexpected,’ ‘expected,’ and ‘serious.’ As our patient population is expected to be ill at baseline (due to their underlying medical conditions and perceived need for RBC transfusion), it is expected that they will have a number of unrelated adverse health events during the course of their involvement in this protocol. Therefore, we will limit the scope of our adverse event monitoring and recording to the following:

1. Serious adverse events (SAEs) will be defined as:

- Death believed to be related to the study procedures, or a death that is unexpected considering the acuity of a patient.
- A life-threatening experience believed to be related to the study procedures.
- Persistent or significant disability or incapacity that is of greater frequency or severity than what would be normally expected given the nature of their medical condition(s).
- An event that jeopardizes the human subject and may require medical or surgical treatment to prevent one of the preceding outcomes and is not expected in the course of their standard treatment regimens.

2. Adverse events possibly related to the study procedures will be defined as:

- Profound anemia (Hemoglobin < 6 g /dL).
- Renal failure requiring renal replacement therapy.
- Myocardial infarction.
- Non-hemorrhagic stroke.
- Mesenteric ischemia (ischemic events secondary to anemia).
- Syncope
- Falls

All adverse events will be indicated on the data forms for the study and on the specific adverse event report forms and all serious adverse events will be reported to the site IRB within 24 hours of the research team learning about the event followed by a more detailed written report to the IRB. The following information about adverse events will be collected: 1) the onset and resolution of the event, 2) an assessment of the severity or intensity of the event, 3) an assessment of the relationship of the event to the intervention, and 4) any action taken because of event. Reporting of SAEs to the IRB will be conducted by the PIs of this application.
