# Supplementary material for: Measuring the impact of ambulatory red blood cell transfusion on home functional status: study protocol for a pilot randomized controlled trial
Source: Trials. 2017 Mar 31;18:153. doi: 10.1186/s13063-017-1873-z (PMC5374599; doi:10.1186/s13063-017-1873-z)
Supplement: Supplementary file 3 — Telephone assessment Data Capture Form. (PDF 12 kb) [file 13063_2017_1873_MOESM3_ESM.pdf]

Week One Phone Assessment

Demographics

Mayo Clinic Number ______________________________

Date of Week One assessment ______________________________

Height ______________________________
(cm)

Weight ______________________________
(kg)

Medications

Were any medications added (including chemotherapy) during Week One?
☐ No
☐ Yes

List medications added during Week One ______________________________

Were any medications stopped during Week One?
☐ No
☐ Yes

List medications stopped during Week One ______________________________

Health/Life Events

Were there any health events during Week One - check all that apply
☐ None

- ☐ Hospitalization

- ☐ Unscheduled physician visit

- ☐ Transfusion at alternate facility

Type of transfusion(s) - check all that apply
☐ RBC

Number of units ______________________________

- ☐ Platelet

Number of units ______________________________

- ☐ FFP

Number of units ______________________________

- ☐ Cryoprecipitate

Number of units ______________________________

- ☐ New illness or injury affecting functional status

Date of new illness or injury affecting functional status ______________________________

Describe new illness or injury affection functional status ______________________________

Were there any other health or life events affecting functional status during Week one?
- ☐ No
- ☐ Yes

Date of other event affecting functional status ______________________________

Describe other event affecting functional status ______________________________

Was there a change in residence type during Week One?
- ☐ No
- ☐ Yes

Date of change in residence ______________________________

Type of residence
- ☐ Home or retirement home
- ☐ Nursing home
- ☐ Other

Describe other type of residence ______________________________

Adverse Events

Adverse events during Week One - check all that apply
- ☐ None

-
- ☐ Profound anemia (Hemoglobin less than 6 g/dL)

Start date of profound anemia ______________________________

Was this an SAE?
- ☐ No
- ☐ Yes

-
- ☐ Renal failure requiring renal replacement therapy

Start date of renal failure requiring renal replacement therapy ______________________________

Was this an SAE?
- ☐ No
- ☐ Yes

-
- ☐ Myocardial infarction

Start date of MI ______________________________

Was this an SAE?
- ☐ No
- ☐ Yes

-
- ☐ Non-hemorrhagic stroke

Start date of non-hemorrhagic stroke ______________________________

Was this an SAE?
- ☐ No
- ☐ Yes

-
- ☐ Mesenteric ischemia (Ischemic events secondary to anemia)

Start date of mesenteric ischemia ______________________________

Was this an SAE?
- ☐ No
- ☐ Yes

-
- ☐ Syncope

Start date of syncope ______________________

Was this an SAE?
- ☐ No
- ☐ Yes

\- ☐ Falls

Start date of falls ______________________

Was this an SAE?
- ☐ No
- ☐ Yes

\- ☐ Shortness of breath

Start date of shortness of breath ______________________

Was this an SAE?
- ☐ No
- ☐ Yes

\- ☐ Chest pain/pressure

Start date of chest pain/pressure ______________________

Was this an SAE?
- ☐ No
- ☐ Yes

Device and Mobility

Was the device worn all week?
- ☐ No
- ☐ Yes

Describe reason why device wasn't work all week ______________________

Was the device charged this week?
- ☐ No
- ☐ Yes

Are there any concerns related to device?
- ☐ No
- ☐ Yes

Describe concerns related to device ______________________

Walking aid used?
- ☐ No
- ☐ Yes

Type of walking aid
- ☐ Crutches
- ☐ Zimmer frame
- ☐ Walking frame
- ☐ Highwalker
- ☐ Other

Describer other type of walking aid ______________________

PROMIS Global 10 Survey

Was PROMIS 10 survey completed?
- ☐ No
- ☐ Yes

In general, would you say your health is:
- ☐ Excellent
- ☐ Very good
- ☐ Good
- ☐ Fair
- ☐ Poor

In general, would you say your quality of life is:

- ☐ Excellent
- ☐ Very good
- ☐ Good
- ☐ Fair
- ☐ Poor

In general, how would you rate your physical health?

- ☐ Excellent
- ☐ Very good
- ☐ Good
- ☐ Fair
- ☐ Poor

In general, how would you rate your mental health, including your mood and your ability to think?

- ☐ Excellent
- ☐ Very good
- ☐ Good
- ☐ Fair
- ☐ Poor

In general, how would you rate your satisfaction with your social activities and relationships?

- ☐ Excellent
- ☐ Very good
- ☐ Good
- ☐ Fair
- ☐ Poor

To what extent are you able to carry out your everyday physical activities such as walking, climbing stairs, carrying groceries, or moving a chair?

- ☐ Completely
- ☐ Mostly
- ☐ Moderately
- ☐ A little
- ☐ Not at all

In the past 7 days How would you rate your pain on average?

- ☐ 0
- ☐ 1
- ☐ 2
- ☐ 3
- ☐ 4
- ☐ 5
- ☐ 6
- ☐ 7
- ☐ 8
- ☐ 9
- ☐ 10

In the past 7 days How would you rate your fatigue on average?

- ☐ None
- ☐ Mild
- ☐ Moderate
- ☐ Severe
- ☐ Very severe

In general, please rate how well you carry out your usual social activities and roles. (This includes activities at home, at work and in your community, and responsibilities as a parent, child, spouse, employee, friend, etc.)

- ☐ Excellent
- ☐ Very good
- ☐ Good
- ☐ Fair
- ☐ Poor

In the past 7 days How often have you been bothered by emotional problems such as feeling anxious, depressed or irritable?

- ☐ Never
- ☐ Rarely
- ☐ Sometimes
- ☐ Often
- ☐ Always

Was subject withdrawn from study during Week One?

- ☐ No
- ☐ Yes

Why was subject withdrawn? ______________________________

Notes: ______________________________
